# Public concerns and attitudes towards autism on Chinese social media based on K-means algorithm

**DOI:** 10.1038/s41598-023-42396-4

**Published:** 2023-09-13

**Authors:** Qi Zhou, Yuling Lei, Hang Du, Yuexian Tao

**Affiliations:** Hangzhou Normal University, 2318th Yuhang Tang Avenue, Hangzhou, 311121 Zhejiang Province China

**Keywords:** Public health, Neurological disorders

## Abstract

To investigate the hot topics and attitudes of autism in the larger community. In this study, we analyzed and summarized experimental texts from the social media platform Zhihu using the TF-IDF algorithm and K-means clustering approach. Based on the analysis of the 1,740,826-word experimental text, we found that the popularity of autism has steadily risen over recent years. Sufferers and their parents primarily discuss autism. The K-means clustering algorithm revealed that the most popular topics are divided into four categories: self-experience of individuals with autism, external views of individuals with autism, caring and stressful behaviors of caregivers, and information about autism. This study concluded that people with autism face more incredible negative emotions, external cognitive evaluations of the autistic group reflect stereotypes, the caregiver’s family suffers high financial and psychological stress, and disorders caused by disease in autistic individuals.

## Introduction

Autism Spectrum Disease (ASD) is a severe neurodevelopmental disorder with an unclear cause and a poor prognosis, marked by difficulties in verbal communication, abnormalities in social interaction, and stereotyped behavior^1–3^. According to the World Health Organization, the prevalence of autism has risen at an alarming rate over the two decades, reaching 6.5 per 1000 persons^4^. Autism prevalence among children in the American Health Interview Survey increased from 1.68% in 2014 to 2.48% in 2017, meaning 1 in 59 persons^5,6^. The Chinese Autism Education and Rehabilitation Industry Development Report estimates that there are over 10 million autistic persons in China, with over 2 million children^7^. Moreover, some researchers find that China may have a higher prevalence of autism due to a shortage of medical workers, public misinformation, and parental concealment, resulting in fewer diagnoses for people with autism in remote areas^8^. Wu’s research also states that the Chinese ASD community is in a neglected position and urgently deserves the attention of the public^9^.

However, most autism-related research has focused on high-income countries, and there is a lack of research on the low- and middle-income countries where most people with autism live^10–12^. Studies have found that public attitudes towards ASD are very different in the US and China, with 86–91% of US citizens showing adequate awareness of autism, compared to 57–65% in China^13^. In addition, researchers have debated the involvement of people with autism in public engagement, with some believing that the illness makes it difficult for the autistic community to engage in public engagement and others believing that people with autism are capable of actively participating in public engagement^14^. However, the different perspectives of the public and professionals lead to different views, so it is essential to investigate the public’s attitudes toward people with autism^15^.

Due to the inclusiveness, social media are now the channel where people express their perspectives on ASD allowing professionals to share their experiences and enabling the public to comprehend crucial information^16^. Researchers demonstrate that television and social media platforms such as Twitter^17,18^, Google^19^, YouTube^20,21^, and Facebook^22^ are now equally the primary sources of information for individuals with autism and their stakeholders. As a result, information disseminated on social media will influence public perceptions of ASD, no matter facts or suggestions. Negative portrayals of ASD in the media may reinforce public misconceptions^23,24^; however, the media may also improve public attitudes^13,25,26^. Some researchers have begun to focus on the analysis of autism data on social media platforms such as Twitter and YouTube in an attempt to raise public awareness of autism^16,18,27^. However, these studies are limited to English-language media, lacking of those on Chinese media.

Researchers have recently applied text-mining algorithms to analyze potential information in the medical field^28–30^. Density-based clustering methods exhibit good robustness against noise and outliers^29^, whereas hierarchical clustering has the inherent ability to determine the optimal number of clusters, thereby ensuring a higher level of stability^30^. However, K-means is more efficient than hierarchical and density-based clustering methods due to its lower computational complexity, especially when dealing with massive text data. Furthermore, the cluster results represented by their centroids provide a more intuitive depiction, facilitating a clearer understanding of the clustering characteristics and underlying topics^31^. Given the vast scale of internet comment texts, this study uses the K-means algorithm for topic clustering. However, the K-means clustering algorithm has the disadvantage of requiring the number of clusters to be defined in advance; therefore, the elbow calculation method is used to obtain the K values in this study. Moreover, by combining PCA dimensionality reduction and TF-IDF techniques, this research effectively captures the distinctive characteristics of text data while simultaneously reducing the dimensionality of the feature space, thereby enhancing the efficacy of clustering.

In summary, this study aims to fill the gap in autism research in Chinese social media, investigate potential autism misconceptions, and raise the public’s awareness and tolerance of autism. The rest of the sections are as follows: the second part is about the methods, which include PCA dimensionality reduction, TF-IDF algorithm, and K-Means algorithm; the third part is about results; the fourth part is discussion; and the fifth part is the conclusion.

## Method

### Study subjects

Considering the need to design the text materials to align with the context of Chinese social media^32^, we collected the replies to questions related to “autism” from Zhihu. These replies were obtained as experimental text. The data collection was completed by February 20, 2022.

### Data collection

In this study, we use Python 3.7 to gather the dataset of autism-related Q&A content, editing time, self-presentation, number of views, approvals, and secondary ratings. To ensure that the dataset data fits the algorithm requirements, we merge the dataset’s data and remove duplicate text and blank fields.

### Data processing

#### Text cleaning

In this study, the experimental text was first processed with noise reduction to remove irrelevant content, such as advertisement and web links, to improve the word separation effect and accuracy. Second, we construct a specialized deactivation word list for the experimental text by combining the deactivation word lists of HIT and Baidu with the initial word classification findings. After loading the user dictionary, we use Python’s Jieba database to separate words while using the deactivated word list to delete the inactive terms.

#### TF-IDF algorithm and PCA decomposition

1. Calculate Term Frequency (TF), which measures the frequency of keywords appearing in the text, divided by the total number of words in the document to prevent bias towards longer documents. 2. Calculate Inverse Document Frequency (IDF), which is obtained by dividing the total number of documents by the number of documents containing the specific keyword, and then taking the logarithm of the quotient to indicate the discriminative ability of the keyword across different document categories. 3. Multiply TF and IDF to generate the high-weighted TF-IDF, which represents the importance of a term based on its frequency in the document and across the corpus.

Principal Component Analysis (PCA) is a dimensionality reduction approach frequently employed to transform high-dimensional data into a lower-dimensional representation. PCA dimensionality reduction can effectively reduce the dimensions of the TF-IDF matrix while preserving the essential data information.

This research implements the function with Sklearn. The procedure is as follows: Initially, transform the words in the text into a term frequency matrix using CountVectorizer. Second, calculate the TF-IDF weight of each word using TfidfTransformer. TfidfVectorizer combines CountVectorizer and TfidfTransformer, enabling direct generation of TF-IDF values. Finally, transform the high-dimensional TF-IDF feature vector space into a lower-dimensional representation using StandardScaler and PCA decomposition.

#### K-means algorithm analysis

**Figure 1:**
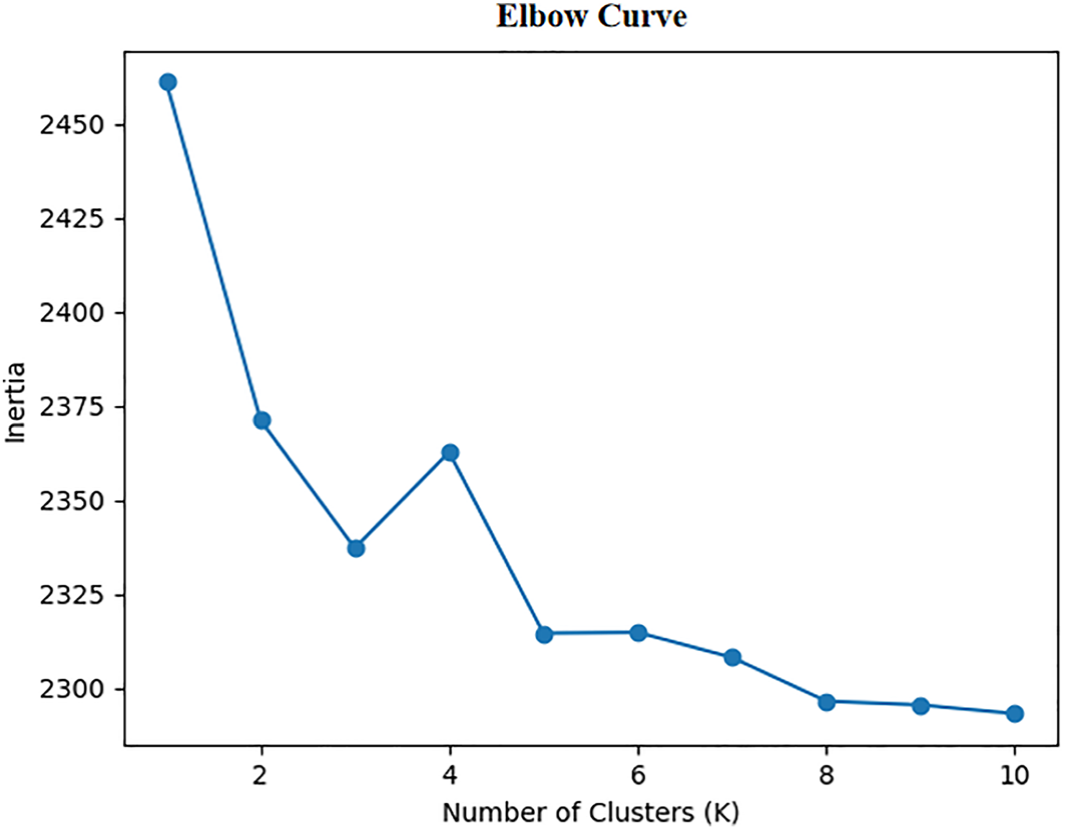
Elbow curve.

The K-means clustering algorithm is a common and well-researched exploratory data analysis technique applied and validated in Song and Wang’s research for Chinese text mining^33,34^. The K-means algorithm enables researchers to understand text data more deeply and uncover counterintuitive insights and patterns. It is particularly beneficial for extracting usable information and knowledge from text data, classifying text data into meaningful groups, and simplifying text data via dimensionality reduction and denoising.The K-means algorithm steps are as follows:

First, Initialize cluster centers: In the KMeansClusterer of the NLTK library, the number of cluster centers can be set using the num_means parameter. This study uses the elbow algorithm to calculate inertia values for different K values to find the perfect cluster number. The inertia value decreases rapidly for small values of K, but the rate of decrease slows down as K increases. We select the elbow curve as the optimal value of K. Secondly, Iterative optimization: This method clusters the data based on a predefined data set and some clustering centers and iteratively optimizes the clustering centers' locations. Thirdly, Termination requirements: NLTK’s K-means clustering is mainly controlled by the REPEATS parameter. This parameter describes the number of times the algorithm should be executed, each with a distinct initial clustering centre^22^. Based on the results obtained through the elbow method and iterative optimization, we conclude that K = 4 better meets the requirements of this research, as shown in Fig. 1.

#### K-Means algorithm result description

We use TF-IDF for feature extraction, which can provide acceptable input data for better K-means clustering results. However, their objectives are distinct. The K-means algorithm itself requires that the TF-IDF weights be normalized. Moreover, the clustering effect of K-means relies on the high-frequency words of individual features and the combined expression of the entire feature vector. Source words ranked low in the TF-IDF high-frequency words may still have a significant impact^35^.

The K-means algorithm yields a source vocabulary and a clustering vocabulary as output. The clustering source vocabulary is the initial vocabulary set to initialize cluster centers. The source vocabulary for clustering is typically a representative set of words whose results can be used as hot topics, whereas the individual clustering vocabularies are sets of words within each clustering cluster that may be more diverse and precise. The vocabulary in the clusters that result from clustering will differ from the clustering source vocabulary, but there is a connection between them.

## Results

### Description of textual material

This study comprised 191 questions concerning autism from the Zhihu Q&A community as of February 20, 2022, and we analyzed the data to create a final experimental text of 4338 comments totaling 1,740,826 words.

## Research trend analysis

Responses posted on the Zhihu platform involve both initial and numerous revisions. This study showed that 75.2% of initial posts and 24.8% of subsequent modifications were updated. Respondents’ increased incentive to add questions is associated with their identification and the duration of autism. This phenomenon also indicates that autism groups require long-term social attention. The increase in the number of topics about autism around April each year may be related to the fact that April 2 is World Autism Day.

### Basic characteristics of Zhihu users

Homemakers, individuals with autism, psychologists, rehabilitation therapists, teachers from traditional or special schools, and social media practitioners are the primary respondents to the autism topic. Homemakers and people with autism are the main posters and editors, eager to share their progress, feelings, interventions, and encouraging comments. Psychologists, rehabilitation therapists, teachers, and social media practitioners use their professional and educational experiences as the basis for information output, actively participate in community discussions and guide people with autism and their families through scientific rehabilitation interventions.

### Basic characteristics of autism topic keywords

After obtaining the results of Jieba word division, the TF-IDF algorithm was used to perform word frequency statistics and mine the keywords in the text to calculate their weights, as shown in Table 1. The results of intelligent topic segmentation based on K-Means clustering analysis are shown in Table 2.

**Table 1:** Top 10 keywords and weights.

Ranking	Keywords	Weighting
1	Autism	0.03862
2	Kids	0.03575
3	Children	0.01452
4	Intervention	0.01293
5	Disorder	0.01084
6	Language	0.01076
7	Ability	0.00962
8	Parenting	0.00903
9	Socialization	0.00737
10	Performance	0.00733

**Table 2:** Cluster analysis results.

Source terms	Frequency statistics	Clustered vocabulary		
		Noun	Verb	Adjective
Autism	26	Emotional life, emotional reactions, reticence, communication, dissociation, glossiness	Cover up, conceal, keep away, control, forced, get better, social interaction, care, criticize	Powerless, stiff, anxious, ashamed, difficult, sad, cruel, sad, tired, depressed, cautious, indifferent, introverted, unhelpful, bewildered, helpless, reticent
Other people	53	Alien, corner, teacher, friend, campus college, fellow classmate, school, poster, TV series, environment, occasion, TV, computer, public, human dignity, society, tone, idiot, monster, world, willingness	Bully, corporal abuse, walk away, withdrawal, submit, punish, reinstate, dump, doubt, influence, taunt, distort, insult, perceive, ingratiate	Aggravated, bad, indifferent, hard, cranky, sensitive, choked, disgusted, strange, twisted, chilled, angry, naive, ridiculous
Child	35	Primary school, children, whole class, regular people, children, childhood, classroom, control, imagination, egoism, older brother and sister	Cry and fuss, run around, treat, integrate, impose chaperone, spend money, trick or learn, weak	Polite, painful, anxious, sensitive, tortured, depressed, familiar, cruel, weak, confused, embarrassed, indifferent, silent
Disorders	8	Barriers, Society, Identification, Intelligence, Worldliness	Train, diagnose, imitate, socialize, express, speak, communicate, lose control	Broken, anxious, struggling, uncomfortable, in pain

## Discussion

This study used cluster analysis to identify four popular topics for user discussion: self-experience of individuals with autism, external views of individuals with autism, caring and stressful behaviors of caregivers, and information about autism.

### Self-experience of people with autism

#### People with autism are more susceptible to negative emotions and are unable to express them

Anxiety, depression, humiliation, sadness, and fatigue indicate the unfavorable orientation of the autistic group’s emotional life and emotional responses. While words like mask, cover-up, away, control, compelled, and powerless indicate that the autistic group is more prone to adopt negative attitudes to cope or not cope with the catharsis of their emotions in response to negative emotional influences. This study indicated that only individuals with mild autism or those who had recovered successfully from scientific interventions actively shared their experiences on the Zhihu platform. During childhood, this group had an active social mindset and did not feel different from other children their age. However, due to unwarranted verbal and behavioral bullying, different worldviews, and ignorance by parents or teachers, autistic individuals may blame the illness for their situation and then magnify the negative emotions caused by the illness, causing them to turn closed off. Terrible childhood experiences increase the probability that autistic individuals will spend their adult lives alone, less socially engaged, and in a world of their creation. To address this problem, we consider that professionals can build and validate autism-related assessment observation scales to recognize emotional changes in autistic individuals, focus on changes in autistic individuals' inner worlds, and support their emotional needs.

#### Individuals with autism continue to express optimism in facing life’s challenges

Words like reticent, apathetic, and introverted reveal the autistic group’s opinion of their situation. Favorable terms such as improvement, social engagement, and communication suggest that the autistic population has a constructive outlook. A small percentage of the autistic population knows they may be weaker than average in learning ability, communication ability, emotional control, psychological tolerance, and even self-care. However, they have a nonchalant attitude and are proactive in accepting rehabilitation interventions to care for their family and friends uniquely. This phenomenon is similar to Uddin and Heselton’s findings in that the autistic group develops resilience when overcoming adversity^36,37^.

### External cognitive evaluation of the autistic group contains stereotypes

Schools, the media, and the general public are the primary sources of external stereotypes concerning autistic persons.

#### The school condition of the autistic people

Words like alien, bullying, aggravation, dreadful, bullying, corporal punishment, walking away, dropping out, in the corner, discipline, school, and reinstatement indicate that school life for the autistic community is not as pleasant. Due to their disease, people with mild autism struggle academically, while those with severe autism are even discouraged from attending school. In addition to the disease’s susceptibility to peer indifference and bullying, some teachers, classmates, and parents of classmates disapprove of autistic children attending traditional schools, fearing they may disturb classroom discipline and the quality of education^38^. The study by Yang Guangxue also shows that teachers cannot give particular care to the autistic group and struggle to deal with the stress and destructive emotions caused by parents^39^. All of the circumstances above compel us to consider how to guarantee the right of autistic children to primary education in traditional schools and how to enhance the special education system for the autistic group. Therefore, we believe that autistic groups in China need a combination of primary and supplemental education for schooling to be effective. Creating traditional and special education campuses must prioritize school environment construction, teacher capacity development, and general parental guidance^40^. We may use the Australian experience in the classroom to focus on creating a barrier-free environment^41^. Teachers actively pursue tenure in autism special education, creating rich autistic curricular resources and systematic special teaching methods based on their general school teaching qualifications^40^. Schools can invite students' parents to autism education workshops to alter their stereotypical view of disability.

#### Mass media in which autism is frequently discussed

Words such as television series, posters, lectures, environments, occasions, and computers indicate that autism is receiving more attention in the mass media. Nevertheless, we observe that the media now portrays autism information negatively. Exaggerating the dangers of autism, stigmatizing autism, and misinforming about autism are instances of biased news that not only make people with autism feel humiliated but also aggravate the public stereotype of the autistic community, generating ostracism and disgust. Many scholars also know that negative prejudice and stigma in media coverage of autism may exacerbate social isolation among people with autism^42,43^. In response to this phenomenon, we believe the media should capitalize on its widespread transmission to play a constructive role. As the primary means by which the general public learns about the autistic community, the mass media should be responsible for scientifically popularizing professional autism information and paying attention to the quality and quantity of their coverage^44^. Professionals in the media should create an excellent online environment for persons with autism, minimize stigmatizing expressions about autism, and protect autistic people’s privacy^13,26^.

#### Public stereotypes of autistic groups in society

Words such as mockery, malice, fool, monster, dump, and distortion reflect the public’s explicit and implicit prejudice and rejection of the autistic group. We find that the general public has a superficial awareness of autism, and their ignorance and preconceptions make it easy for them to feel scared when interacting with autistic groups. The cause of this phenomenon might be misinformation from the media or because autism has recently attracted attention in our country^45^. Therefore the correct perceptions and impressions have not yet formed. In addition, the stereotyped influence of the social public on people with autism is reflected in a vast social gap, which restricts several rights of the autistic group, including work, education, and medical care. One researcher surveyed the public’s perceptions of the autistic community. Sixty-five percent of those polled believe society is unwilling to accept the autistic group and are aware of job discrimination against this group^46^. Therefore, we should respond to the United Nations' initiative to educate people from all social classes about autism, eliminate prejudice and stereotypes against autistic people, and provide autistic individuals with equal access to employment, education, and medical care.

### Financial and mental strain, self-stigma, and external stigma exist for caregiver families

#### Families of caregivers experience both financial and psychological strain

Words such as reality, self-care, accompanying, tormenting, directing, intervention, treatment, problem-solving, diagnosis, and evaluation indicate the overwhelming economic and psychological pressure on parents and family members to care for the autistic group. Parents must accompany their children to expert rehabilitation interventions and pay for expensive, long-term rehabilitation instruction. The situation is consistent with Cristiane et al.’s research, which found that parents in the typical autistic family struggle to balance job and family responsibilities and that autistic families in underprivileged areas may even stop getting treatment due to the cost^46–48^. The essence of the status quo is that most autistic families in China are currently in the self-help stage, and only a small number of families are eligible for outpatient reimbursement of 70 percent^49,50^. As a result, we must improve the social support system and develop more socialized and private rehabilitation institutes to serve a larger Population. On a psychological level, the lack of knowledge about the daily life care and professional rehabilitation of the autistic group puts a higher burden on parents. As parents age, they are anxious about spreading the stress of daily care to other family members. In addition, parents are concerned that their lack of experience and access to the most recent information about the illness might impede their children’s rehabilitation^51^. The situation agrees with the findings of Wang et al. We claim that through the engagement of relevant national policies, we must develop a service system for the caregiving obligations of the autistic group and provide caregivers with psychological support in the form of social solidarity^52^. Let the state shoulder the rehabilitation training system for autistic groups, and alleviate a portion of the load of autistic caretakers^53^.

#### Self-stigma and collateral stigma among caregivers

Words such as genetic, neurological, root cause, and avoidance are linked with autism to reflect the self-stigma of parents who blame themselves for their children’s suffering and assume they caused the illness. We find this phenomenon results from unknown etiology and causes. Without a scientific explanation, parents are reluctant to admit their child has a disability and blame themselves. The concept of karma also contributes to parental guilt about their children’s misfortunes^50^. Words linked with autism, such as sociability, embarrassment, commoner, low self-esteem, and inclusion, reflect social discrimination and caregiver stigma may induce collateral stigma. This phenomenon necessitates researchers to consider the interaction between individuals and societal groups. Caregivers of individuals with autism may experience social exclusion and isolation, resulting in significant psychological pressure and their voluntary or subtle marginalization by society^54^. Kevin et al. found that self-stigma and linked stigma were positively associated with parental symptoms of depression and anxiety, which led to social pressure and isolation^55,56^. Corcoran et al. even concluded that parents of autistic children are more likely to suffer from emotional disorders^57^. We observe that professionals and the public must avoid stigmatizing caregivers. Professionals must be able to rapidly identify parental confidence deficits, emotions of despair, and social isolation and provide practical skills such as self-compassion and positive thinking approaches by constructing long-lasting support systems^58–60^. The public should also reflect critically on the correct attitudes, perceptions, and behaviors necessary to eliminate discriminatory behavior^61^.

### Barriers encountered by individuals with autism

People with autism have severe social and language impairments, and according to cluster analysis, the words connected with autism are impairment, social, training, socialization, expression, speech, imitation, and communication. Xiao Fufang et al. concluded that the autistic group exhibited adverse reactions to social impairment, language impairment, and stereotypical behavior^62^. However, the results of this study showed that the autistic group did not actively mention the presence of stereotyped behavior. We suggest that the phenomenon’s cause may be related to the ability to actively share their experiences on Internet platforms, which is mild autism. It is also likely that they need to gain knowledge of people with autism and their parents to accurately identify stereotyped behavior as a dull habit. Some autistic people are conscious of their stereotypes and are compelled by societal pressure to imitate others intentionally^60^. We believe that in the early diagnosis stage, focus on screening children with family history and pay attention to premature attention disengagement, repetitive behaviors, and other risk signals in children^63,64^. In the later rehabilitation period, the government can rely on the community and schools, families, and institutions to establish an autistic support system^65^.

## Conclusion

On the Chinese Internet, acceptance and awareness of the autism community are gradually growing. We must address the appropriate medical and employment demands of autistic individuals, pay attention to poor autistic families, and establish a nationwide autism support system. Professionals and the media should contribute to the distribution of accurate autism information.

## Data Availability

The data that support the findings of this study are available from The Zhihu platform but restrictions apply to the availability of these data, which were used under license for the current study, and so are not publicly available. Data are however available from the authors upon reasonable request and with permission of The Zhihu platform. Please contact the corresponding author Tao if you want to request the data from this study.
